# Livelihood Risk, Culture, and the HIV Interface: Evidence from Lakeshore Border Communities in Buliisa District, Uganda

**DOI:** 10.1155/2019/6496240

**Published:** 2019-05-16

**Authors:** Japheth Nkiriyehe Kwiringira, Paulino Ariho, Henry Zakumumpa, James Mugisha, Joseph Rujumba, Marion Mutabazi Mugisha

**Affiliations:** ^1^Kyambogo University, Department of Sociology and Social Administration, Uganda; ^2^Makerere University, School of Public Health, Uganda; ^3^Makerere University, Paediatrics and Child Health Department, Uganda

## Abstract

**Background:**

While studies have focused on HIV prevalence and incidence among fishing communities, there has been inadequate attention paid to the construction and perception of HIV risk among fisher folk. There has been limited research with respect to communities along Lake Albert on the border between Uganda and the Democratic Republic of Congo (DRC).

**Methods:**

We conducted a qualitative study on three landing sites of Butiaba, Bugoigo, and Wanseko on the shores of Lake Albert along the border of Uganda and the Democratic Republic of Congo. Data were collected using 12 Focus Group Discussions and 15 key informant interviews. Analysis was done manually using content and thematic approaches.

**Results:**

Lakeshore livelihoods split families between men, women, and children with varying degrees of exposure to HIV infection risk. Sustaining a thriving fish trade was dependent on taking high risks. For instance, profits were high when the lake was stormy. Landing sites were characterized by widespread prostitution, alcohol consumption, drug abuse, and child labour. Such behaviors negatively affected minors and in many ways predisposed them to HIV infection. The lake shore-border heterogeneity resulted in a population with varying HIV knowledge, attitudes, behavior, and competencies to risk perception and adaptation amidst negative masculinities and negative resilience.

**Conclusion:**

The susceptibility of lakeshore communities to HIV is attributable to a complex combination of geo-socio, the available (health) services, economic, and cultural factors which converged around the fishing livelihood. This study reveals that HIV risk assessment is an interplay of plural rationalities within the circumstances and constraints that impinge on the daily lives by different actors. A lack of cohesion in a multiethnic setting with large numbers of outsiders and a large transient population made the available HIV interventions less effective.

## 1. Background

Human Immunodeficiency Virus (HIV) prevalence in African fishing communities including fish catchers, processors, and traders is much higher (3–4 times) than the national average [1–3]. Of the estimated 2.1 million annual HIV infections globally, majority are in sub-Saharan Africa (SSA) among high-risk populations including fisher folk and lakeshore populations [4]. Across Africa, fishing communities generally live in environments with limited social amenities [5–7]. This is true in Uganda and perhaps worse in the Democratic Republic of Congo (DRC) [8]. Due to persistent internecine conflict particularly in Eastern DRC [9, 10], the Congo has been referred to as a collapsed or an absent state [10, 11]. There are no known studies documenting the situation of fisher folk, HIV, and AIDS in the DRC. What is well known is that people in DRC have suffered enormous loss of life and livelihoods, compounded by institutional failure, loss of national assets, and economic decline [10]. There are widespread poverty and poor health, and education, water and sanitation, and transport are all considered fragile and insufficient [12]. The main limitations to livelihoods development are also due to the exploitative governance system, lack of security, and basic services. In East Africa, there is a high prevalence of risky sexual behavior on landing sites compared to the general population [13, 14]. Fishing communities are among population groups that are most at risk of HIV infection [15–17].

Fishing, as an activity, has been associated with frequent movements which increases the number of sexual partners [18]. Sexually active migrant men have been reported to have multiple sexual partners [19, 20]. Fishermen are often away from home for long stretches of time and, in some cases, there is extended migration which contributes to behavioral changes such as establishment of new sexual relationships [21, 22]. Fishing communities have also been reported to have fatalistic attitudes [23]. Fatalism is the tendency to perceive and believe that there is nothing one can do to change an outcome, therefore one's choices really not mattering, and that human beings are powerless to change outcomes in life [2]. This is linked to high HIV prevalence [24]. For instance, along the Lake Victoria basin in Uganda HIV prevalence is up to three times higher than that of the general population [2, 25] due to sociodemographic, behavioral, and biological factors [26]. In many parts of Africa, uptake of HIV testing by men remains low in high prevalence settings [27]. The HIV-1 prevalence among fishing communities on Lake Victoria in Uganda revealed low prevalence in women than men [28]. The prevalence of HIV among fishing communities on Lake Victoria has partly been attributed to heavy alcohol use [29] and other risk taking behaviors among fisher folk shaped by perceptions and attitudes towards risk. The risk perceptions and attitudes to HIV have been theoretically linked to other risky behaviors associated with fishing such as smoking, illegal fishing, and alcohol abuse [30]. Risk perception has been explained to be a social process [31–34]. People's actions are largely determined by social aspects and cultural adherence [35] to what is expected, what has been experienced, and what is considered normal (customary or traditional) and approved in the community [36]. The role of contextual factors also features in the works of Parsons and Atkinson on the lay construction of genetic risk [37].

Studies on HIV prevalence and incidence among fishing communities have focused on communities along Lake Victoria [14, 21, 38–40]. However, there has been little reported research with respect to communities along Lake Albert which borders the DRC. Despite high levels of awareness about HIV and AIDS in Buliisa district, sexual and reproductive health practices among fisher folk and cross border populations are risky. We explored the major drivers of HIV transmission including the interface of livelihoods, culture, and the availability of HIV prevention and care services in Buliisa district on the shores of Lake Albert in Uganda.

## 2. Theoretical Framework

Although cultural theory has been in use for a longtime, it still offers a pragmatic and broad framework of addressing many questions related to risk, like HIV health-risk behavior. The theory posits that “total knowledge” would be necessary to understand the risks that individuals face [41, 42]. Cultural theory is a way of interpreting how and why individuals form judgments about danger and threat [33, 43–45]. Using this approach, the attitudes and perceptions about risks among fisher folk are determined by the sociocultural relationships within the fishing community, especially that fishing is a subculture [46] of risk-takers living within hypermasculine and sexualized normative environments that promote sexual risk behavior [3, 47]. Risk is a social process and a product of shared beliefs and values [42, 48]. The central assumption of the theory is that there is a relation between modes of social organisation and responses to risk [49].

## 3. Methods

A qualitative study was conducted in Butiaba, Bugoigo, and Wanseko on the shores of Lake Albert along the border of Uganda and DRC. Data were collected from 12 Focus Group Discussions and 15 key informant interviews. Separate focus group discussions of 8-10 participants were held with men and women while in-depth interviews were held with key informants including health workers (Village Health Team members -VHT), staff at health centres; religious and local government leaders, political and technical staff in the health and fisheries departments at the district especially the Beach Management Unit (BMU). Data were analysed manually using content and thematic approaches to identify manifest and latent content in the discussion and interview scripts [50, 51]. FGDs and interview scripts were read several times to identify emerging themes and subthemes. The identified themes and subthemes were used to code data. Subgroup analysis was done involving examination of themes and subthemes in relation to FGD data and key informants. Data coding began during data collection and went on until after data collection. This enhanced continuous analysis while also serving as an analytic method for coding and analysis [52, 53]. The coding process was heuristic and served as an exploratory technique because of this systematic approach; the coding was not just labelling; it was cyclic, from the data to the idea and from the idea to the data [52, 54]. Direct quotations from focus group discussions and interviews were used to present the findings.

## 4. Ethical Issues

The study protocol was reviewed and cleared by Buliisa district local government administration Health Department, Fisheries Department, and the Chief Administrative Officer (CAO). We further informed the subcounty, parish leaders, and the respective beach management units prior to data collection. Due to high illiteracy levels among the study population and a great desire for anonymity, we obtained verbal [55, 56] consent from participants. The refusal to document consent (by either thumbprint or signature) was due to some participants' illegal immigration status and involvement in illegal fishing. Even then, we made sure that key ethical issues pertaining to the research such as its purpose, potential benefits and demands, and methods were communicated and understood. This approach enabled voluntary participation. Before data collection, we ensured that all the three conditions of consent, namely, sufficient information, adequate understanding, and voluntary participation [57], were met. Insisting on written consent and risking recruitment of inappropriate study participants, and losing the trust of participants we opted to obtain verbal consent [58].

Study participants' identifiers were not recorded in transcripts. To ensure that participants did not take part in multiple focus group discussions, a study participants' tracking form was used to record name, age, and village of FGD participants. These lists were destroyed at the end of the data collection to maintain anonymity. The need for confidentiality was emphasized during training of research assistants and applied to conduct the study. We held a debrief meeting at the end of data collection with district and local officials to give feedback on emerging issues.

## 5. Results

We present results following the major identified themes, namely, (i) risk as a way of life, (ii) high risk of fishing and HIV and AIDS perceptions, (iii) gendered livelihood strategies and their influence on risk behaviors, (iv) coexistence of limited HIV services and negative community attitudes and beliefs and, (v) child labour and the risk of HIV infections.

### 5.1. Risk as a Way of Life

Fishermen live in clusters of high population density without privacy. This was due to the mode of settlements and the nature of work weaved with redundancy, alcoholism, music, prostitution, and a latent high-risk skill. Other than alcohol, drugs, and prostitution, the fisher folk have few entertainment options. Fishermen reported having faced many life-threatening and traumatizing situations such as raging lakes, fishing out bodies of fishermen who had died, survived capsized boats and canoes without life jackets, and staying out in the cold throughout the night and sometimes when it is raining; these and more such scenarios made fishermen get used to risk taking. As such, HIV and AIDS to fisher folk were* “farfetched fears*” because the mood was close to* “we may die tonight…”* Life on the lake shore and along the border was an environment of* “easy come; easy go.”* In fishing trade, inputs were direct and clear and returns instant. This was unlike other livelihoods especially farming where there is a long time-lag. In contrast, after a fishing expedition (that can sometimes be risky, but also more profitable), once a boat docked, buyers would bid for fish even before the fisherman set foot on dry ground. This income cycle happened averagely every twenty-four hours.

### 5.2. Livelihood Risks and HIV Perceptions

In a fishing context, without* reasonable* risks, no money can be earned. For instance, fish fetches the best prices when the lake is volatile. Fishermen dare and go in the lake and when they come back they earn a fortune. Therefore, the introduction of HIV as a life-threatening epidemic or risk to be avoided was not different from the other risks that the fisherman is used to within the fishing trade. A veteran fisherman at Wanseko put it thus:

*Yes, you need to cast your net deeper in the lake and among the community to catch the best women: time and life are short. A storm may destroy all your wealth and life just like that; so why not live to the full since no one is sure?”* FGD Wanseko

*“AIDS is not our problem here, what we need is better fishing equipment and boats so as to earn more money. If you have money, AIDS cannot kill you.”* [FGD Wanseko]

The regular cash income, irregular working hours, and being away from family and spouses for prolonged periods of time made fishing a high-risk livelihood. The demands of the fishing livelihood encouraged the consumption of drugs and prostitution with women seeking income opportunities along landing sites to sell food, alcohol, and sex. During seasons of low catch, women tended to trade sex for fish. This put women at risk of exploitation and constrained their ability to negotiate for condom use. These circumstances increased their exposure to HIV. Fisher folk ranked HIV lower (in terms of risk) to the perils of fishing which presented them with fast death on the lake. Fishing was thought to be an ultra-risk activity, more risky than unprotected sex, and therefore catching HIV was not something to worry about. Lake shore communities extrapolated the risk culture in the fishing trade to sexual practices including noncompliance to safe sexual practices. Fishermen perceived their occupation as more life-threatening compared to the risk of HIV.

“How can a veteran fisherman who has survived crocodiles, capsizing, storms and winds; swam in the lake the whole night, be scared of a mere sickness? Just an infection!? You are all cowards and you will die before me.” FGD Participant.

In contrast to marine risks, fishermen argued that HIV does not kill anyone overnight while the lake could do so in seconds!

### 5.3. Gendered Livelihood Strategies and Their Influence on Risk Behavior

Settlements among lakeshore communities were shaped along livelihoods. At family level, a clear division of labour was noticed with husbands involved in fishing at the lake; wives stayed at Ngwedo (*a fertile island where food is grown*) with children staying at home. None of these groups had complete socioemotional support in form of love, care, and intimate interaction including guidance and counselling. For most parents, the little income the children earn is seen as more important to the parents than the opportunities through education.

When you go in the village, the situation is very bad; people are initiated into sex very early while everyday people embark on drinking and pleasure seeking as early as 9am in the morning. [Religious Leader, Buliisa Town]

It was reported that young girls who ventured into fish vending ended up in sexual relationships with drug dealers and other smuggling groups leading to teenage pregnancies which are closely associated with increased HIV risk. This situation is worse for orphaned and vulnerable girls because they have no source of reliable livelihood. The study sites were characterized by many strangers that were also transient. As a result, the population had varying levels of HIV knowledge, attitudes, behavior and competencies to risk and masculinity perceptions, stigma, and Prevention of Mother to Child transmission (PMTCT) of HIV. The cultural practices such as the acceptance of multiple sexual partners as a measure of an* accomplished fisherman* coupled with the low knowledge about HIV among the Alur from the DRC had posed a serious threat to community sensitisation about HIV prevention. The following excerpts highlight the gender–livelihood intersections and HIV risk among the lakeshore communities.

*“…you mean you are here to stop us from eating things and enjoying ourselves? You can go elsewhere; for us we have lived with AIDS for more than 20 years now and nothing has happened. I have lost six wives and now I have two. If you can; talk to women but leave us (men) alone. I have gone to make money and to see my lovers; talk to those ones who are idle.” FGD* Bugoigo

*Sex is our hobby here, fishermen love women; and women from Congo know this! We cannot survive without refreshing ourselves with women……! FGD Men Wanseko*

*“For us fishermen, we tend to move a lot and we are mobile; you might decide to cheat on your wife when you are away…….this is how most of us do it.” FGD Men Wanseko*

*“I am still young and I can change ladies like clothes so long as a lady is beautiful I don't mind….! “Everywhere I go I need someone to comfort me.”* Young Fisherman Butiaba

Married women were also likely to be divorced if the husband discovered that a woman was HIV positive.

*“…I am reluctant to take an HIV test. Suppose I get positive results, my husband will blame me even when he is the one that has many sexual partners…he thinks it is his right to have many partners since he has money…”* Young Female, Wanseko

### 5.4. The Nexus of Limited HIV Services, Negative Community Attitudes towards Service Use, and HIV Risk

Services targeting fishing communities were limited because of the difficulty in mobilizing and reaching transient communities. This posed challenges in targeting for HIV prevention services since high mobility linked the high prevalence and low prevalence areas. The high prevalence areas (particularly across the border in the DRC) were also poorly serviced and had low HIV knowledge and competencies. Majority of the respondents had not tested for HIV due to misconceptions of being safe as one migrant fisherman retorted: “... I* have never tested because I know I do not have AIDS”*. In some cases, the risk of HIV infection was disregarded especially when people had known each other for a while:

*“Having known someone for so long and you've always had feelings for them; when an opportunity strikes you don't hesitate… you may lose your only chance…you prefer to live on this memory….”* Fisherman Butiaba

Condom use was not popular within the fisher folk due to myths and negative attitudes related to their use coupled with meanings attached to a live sexual act.

*“………condoms make sex artificial; AIDS came for men like me.”* (Men,FGD Bugoigo)

*“You cannot use condoms with your wife with whom you have stayed for so long; if she wants to use condoms, let her go away.”* (Men,FGD Wanseko)

*“I don't trust these condoms these days. These condoms don't allow all the sweetness … you cannot enjoy freely.”* Men FGD Bugoigo

*“I have slept with over 35 women and each time I have not used a condom because of the need to feel the sweetness of sex in each of them. Without a condom things are original!”* (Local leader,BMU)

Condom use was equated to eating* headless fish*. Headless fish meant that one would not know the* type of fish* they had eaten.* “You only know the fish from its head; this is why we do not use condoms.” *The epistemology of fish was represented by the concept* Kitwe,* which in local parlance means head. However, in the context of HIV,* Kitw*e literally means unprotected sex. The* Kitwe* syndrome allowed the privileging of unprotected sexual encounters over safer sexual practices despite the risks involved. The sexual experience, particularly its* sweetness* and* liveliness,* was considered more important than safety. This was the social context of HIV risk among the study population.

*“There is no time for a condom; we want things as live and fresh as possible. You see we are used to fresh fish…. So everything has to be that way! We normally eat kitwe..!”* (Men,FGD Wanseko)

*“My husband cannot allow using condoms…. he says that it is not the reason he paid bride price.”* (Married Woman,Bugoigo)

It was believed that condoms interfere with* meaningful* sex, with men controlling terms of sexual encounters. This increased susceptibility to HIV risk in turn increased HIV risk acceptability. In many cases, unprotected sex defined relationships as* serious* and* permanent.* There was a symbolic love value in unprotected sex especially* commitment*. A* sexual experience* was more important than* sexual safety*. The perceived benefits of unprotected sex included pleasure, proof of commitment, love, trust, and an enduring relationship based on sacrifice. This commitment had an intrinsic value with various meanings such as empathy and* one blood-my blood*.

Respondents did not perceive themselves as being* at risk* of HIV infection because they had not been to* far off *places. This implied that only those who had gone* to far-away places* were at risk of HIV infection. Proximity to a sexual partner was equated to sexual safety. This limited demand for HIV services especially testing and condom use. Those that had sex with people they knew perceived themselves as not being at risk, especially if that person* looked healthy*.

*“...if she looks beautiful and is healthy, with big eyes, with a smooth -tender skin and happy; then she's O.K.…it's better to drown in an ocean than stagnant water…if you're to eat a frog, eat a fattened one…!”* (Fisherman FGD Wanseko landing site)

The social desire to conceive was highly valued by both sexes as a consummation of masculinity, femininity, and a relationship. The procreational value of sex was linked to parenthood. Because of this, those that wished to have children were not likely to use condoms. Value judgments and perceptions of the sexual partner encouraged complacency, which presented a fertile environment for the spread of HIV.

### 5.5. Child Labour and Risk of HIV Infection

Child labour and household poverty had also exposed children to the risk of HIV infection through high-risk behaviors. In some cases, children as young as 10 years were already involved in fishing. Instances of some school going children catching small fish for sale during break time were noted. Child labour was viewed by some parents and community leaders as economic empowerment and a viable livelihood base for children especially where the returns on fishing had proved worthwhile in meeting basic needs. Child labour was contrasted with the many years in school while providing no certainty of getting a well paying job. Even when this onset of income earning opportunities for children led to high school dropout rates, to many households fishing was a rational choice.

*“Parents tend to neglect their children and abdicate their responsibilities... There is an I-don't-care attitude in a widely permissive society. Children do not seem to belong to parents anymore but belong to peers that are driven by emotions and excitement. This is the trap that these young people find themselves. The small income that a child earns seems to be more important and critical than the child's future to most parents here.”* KI religious leader

*“Defilement, rape and early marriages are very high here although not reported. Drugs are seen as morale boasters to stave off cold, isolation and boredom. You see, even young girls do drugs. Girls are a key factor in drug dealing especially from Congo. Young girls hoodwink security and play...once they get high on drugs, then no one wants to know there is HIV.”* KI Religious Leader.

Generally, the fishing industry did not support vocational skills training or formal education; this resulted in a majority of youth being trapped in income earning activities. Yet, these activities were not sustainable due to lack of life and business skills which posed a threat to majority of the fisher folk including exposing them to HIV and AIDS related socioeconomic and health challenges once they got infected and their health degenerated.

## 6. Discussion

Risky sexual behaviors were wide spread and attributable to the risky life style compounded with vulnerabilities at the lake [59, 60]. A study conducted in Kenya found that fishermen acquired other female sexual partners because they were often away from home for long stretches of time in search of fish [21]. Abdication of parental roles led young people to rely on peer influence and the attendant pressure. It has been documented that separation of children from care givers results in several personal troubles including initiation into adult lifestyles including sex at an early age [37]. Puffer et al. [61] report that lower caregiver monitoring results in high-risk behaviors. The early access to income initiated children into adult behaviors such as alcohol consumption, addiction, and sexual relationships. This finding is in line with the assertion that young men after earning disposable income engage in unprotected sex [62]. There is evidence that male youths spend some earnings to initiate sexual relationships [61].

Extended migration contributed to behavioral responses such as the establishment of new sexual partnerships which not only expanded fishermen's sexual networks, increasing their likelihood of encountering an HIV positive sexual partner, but also enhanced their role as bridges for disease transmission, linking both fishing and nonfishing communities around the lake. The primary concern of the fisher folk centred around their survival on the lake, rather than taking preventive measures against HIV infection [2] which thrived within a belief that good things come to those who risk and not to cowards. While assessing the burden of HIV and utilization of HIV prevention and treatment services, studies [5, 63, 64] reveal that one of the reasons HIV prevalence remains is the engagement in high-risk life styles and high mobility [6].

Poor service delivery did not encourage HIV testing which was central to both prevention and care. Combined with poor attitudes, service gaps did not motivate people to go for routine HIV counselling and testing (RHCT) even when mobile services were sometimes brought to the area by agencies such as the Aids Support Organisation (TASO). This was further complicated by irregular HIV services due to the peripheral nature of the landing sites. It was reported that services were only available for a few hours on specific days and in a few places. This discouraged HIV healthcare seeking. This complexity was a latent driver to HIV across the lake shore community. This point towards an eminent needs mass sensitisation among the fisher folk on the need for HIV testing even when there are no visible indications that one could be living with HIV. This is consistent with Tumwesigye et al. [6] who found that the coverage of HIV services especially ART among fisher folks was below the national average. Negotiating safe sex was neither always possible nor the best option; there were gender undertones where women were more prone to violence in their attempts to negotiate for safe sex [65, 66]. On the other hand, risk perception was socially organised, with epidemiologically risky behaviors such as sex tourism viewed as mundane activities at a community scale [16]. This was the social construction of risk [62, 67].

Seasons of low fish catch increased risky sex especially among women due to poverty which limited their ability to negotiate condom use. This rhymes with Lubega et al. [68] who attributed failure to use condoms to the need for money that deprives women of the social bargaining power for protected sex. Studies on fishing, gender, and HIV [69, 70] report that the “sex for fish” relationships at landing sites increased during periods of limited supply of fish. Not having sex with a fisherman meant not getting access to any fish and this increases risks of engaging in unprotected sex. In Nyanza, it was reported that, during periods of low catch, fishermen gave preferential access to fish, larger quantities of fish, or simply a guaranteed supply of fish to women with whom they were having sexual relationships [21].

HIV and AIDS activists were perceived as HIV positive or having a relative or friend who is positive. As such, their messages including sensitisation on HIV testing and enrolling in HIV care were not taken seriously. This implies a need to address such misconceptions through comprehensive education and behavior change campaigns for the population at grassroot level. Identification, training, and involvement of community resource persons such as peer educators, expert clients, and leaders in behavior change and community education activities within the local community have potential to improve intervention effectiveness.

HIV risk was also mediated by power relationships [71], familiarity with risk taking, and benefits of unprotected sex. The short term benefits, such as sexual experience and pleasure, monetary gains, spouse and sometimes social approval, avoiding conflict, and rejection, outweighed the benefits of safe sex. Just like all relationships, sexual relations were also power relations [72]. Previous studies have shown these attributes in high-risk groups and settings [73–75] as well as the value attached to unprotected sex [76]. Unprotected sex was seen as a life bond and was perceived as more reassuring and safer than one partner merely “surviving” the infection. Being sexually aware and cautious of the risks involved was not as appealing as being committed to a lover in all situations. Commitment in many ways meant throwing caution away if the couple* loved* each other. “Real” sex [76] and the relationship itself were more important than sexual safety. The notion that risk increased returns seemed to apply when women were beautiful and seemed elusive. The apparent safe sex measures attempted by women were the more reason men were willing to take on more risks to get them. The more a female put up a case for safe sex, the more the man was motivated to take risks, for more pleasure, than safety, with–less-pleasure. Condoms were seen as spoilers of the* real thing*. Physical and nonphysical persuasion were understood and closely associated with coercion [60] including threats and intimidation. Sometimes women gave in to unprotected sex as a means to distinguish between intimate and* nonintimate* partners. All these pointed to unequal negotiation [77]. Because of a high likelihood of gender based violence [78, 79], initiating safe sex negotiations was sometimes perceived as carrying with it greater risks than unprotected sex, at least in the short run.

Therefore, informed choice is not necessarily risk-free or safe. In some ways, “choosing” risk was a form of rationality and perceived safety at least in the short run. To an extent and in context, “wrong” health choices can be* rational* choices, while safe sex choices can be seen as “Irrational” in the circumstances. As such, “risk” varies from one context to another, including the types of social norms, relationships and social networks. The perception and calculation of risk among fisher folk and lake shore communities was within the context of high-risk living with HIV being no exception. Even with HIV risk being imminent,* doing* risk was normal. This was the result of a socialized habitation of risk. Therefore, socioeconomic dimensions of life (of which livelihoods are an important part) affect the nature of HIV risk in a population. Social norms affect behavior, attitudes, practices and interaction [80]. As such, analysis ought to shift from individuals to social factors [81, 82] that shape choice and behavior [37, 83, 84].

## 7. Strengths and Limitations of the Study

The use of qualitative methods facilitated an in-depth understanding of intersecting realities of livelihoods, culture, and HIV risk among fishing communities. Our findings are relevant in informing policy and shaping interventions among high-risk populations both among border and fisher folk communities. The study shows the role of context and livelihoods (poverty or welfare) as both drivers and factors in curbing the spread of HIV and AIDS. Even then, our findings should be interpreted in view of the following limitations: the study covered only one rural lake shore site; thus the applicability of our findings to nonrural, nonborder fishing communities may be limited. However, the fact that our findings resonate well with what has been documented in other fishing communities is reassuring that these findings are credible.

## 8. Recommendations and Conclusion

There is need to design and deliver comprehensive and consistent HIV services that are accessible for the lakeshore communities. Such services ought to be run using various strategies including engaging local champions such as peer educators selected from among the local population especially the fisher folk. Mass campaigns should be intensified during seasons of low fish catch as this period has increased risky sexual behavior. It is important to introduce “AIDS competencies” among the lakeshore community along a model of partner networking and collaboration through training and capacity building for networks beyond the most at risk populations to be more inclusive and responsive. In addition, support has to be extended to those that are HIV positive to encourage ethical conduct in addition to supporting orphans and vulnerable children who in the search for survival fall prey to HIV infection. These interventions have to take into consideration the potential lack of cohesion in a multiethnic setting with large numbers of outsiders and transients. Ignorance, myths and fatalistic tendencies need to be addressed through offering skills training for instance through Functional Adult Literacy (FAL) and other vocational skills training (VST) arrangements that address the Sexual Reproductive Health (SRH) challenges in a tailor made manner. The oil exploration activities in the Albertine region in Uganda and the social mobility associated with a change in livelihoods ought to be closely understood by HIV service agencies in order to plan and respond to the HIV challenge in the area.

Fishing and lakeshore living involve a lot of risks and the profits that accrue from fishing increase this risk. This thinking was extended to sexual and reproductive health. The more it seemed risky, the more they thought it worth trying. There was complacency to general life driven by exaggerated and oppositional forms of masculinity that sometimes challenged conventional medical norms including HIV counselling and testing. The susceptibility of lakeshore communities to HIV is largely attributable to a complex combination of biological, socioeconomic, and cultural factors which converge around fishing activities and communities. Rather than being individual experiences, risk and danger were socially perceived and organised. There is a relationship between how social structures fashion and shape risk in individual lives and how individuals act in response.

Methodologically, the contingent nature of negotiating and maintaining consent during research is crucial in the study of complex, peripheral, transient, and informal populations. In this study, we found that the variability of consent demands reflexive approach where a formulaic devotion to procedure may prove inadequate or even counterproductive [85, 86]. Therefore, different approaches to informed consent are possible. Ethical research can be conducted in various ways beyond merely adhering to orthodoxies. We concur with Humphries and Martin (2000) [87] and Benhabib (1992) [88] that a research process that is not reflexive in the securing of consent shies away from active engagement of research ethics.

## Data Availability

Raw data is not publicly available. All data containing relevant information to support the study findings are provided in the manuscript.

## Abbreviations

AIDS: Acquired Immunodeficiency Syndrome
DRC: Democratic Republic of Congo
FAL: Functional Adult Literacy
HIV: Human Immunodeficiency Virus
SRH: Sexual Reproductive Health
RCT: Routine Counselling and Testing
VST: Vocational Skills Training.
